# High-throughput nanofluidic real-time PCR to discriminate Pneumococcal Conjugate Vaccine (PCV)-associated serogroups 6, 18, and 22 to serotypes using modified oligonucleotides

**DOI:** 10.1038/s41598-021-03127-9

**Published:** 2021-12-09

**Authors:** S. L. Downs, S. A. Madhi, L. Van der Merwe, M. C. Nunes, C. P. Olwagen

**Affiliations:** 1 South African Medical Research Council, Vaccines and Infectious Diseases Analytics Research Unit, School of Pathology, Faculty of Health Sciences, University of the Witwatersrand, Johannesburg, South Africa; 2 Department of Science and Technology/National Research Foundation, South African Research Chair Initiative in Vaccine Preventable Diseases, Faculty of Health Sciences, University of the Witwatersrand, Johannesburg, South Africa

**Keywords:** Laboratory techniques and procedures, Infectious diseases

## Abstract

Current real-time high-throughput Polymerase Chain Reaction (qPCR) methods do not distinguish serotypes 6A from 6B, 18C from 18A/B and 22F from 22A. We established a nanofluidic real-time PCR (Fluidigm) for serotyping that included Dual-Priming-Oligonucleotides (DPO), a Locked-Nucleic-Acid (LNA) probe and TaqMan assay-sets for high-throughput serotyping. The designed assay-sets target capsular gene wci*P* in serogroup 6, wci*X* and wxc*M* in serogroup 18, and wcw*A* in serogroup 22. An algorithm combining results from published assay-sets (6A/B/C/D; 6C/D; 18A/B/C; 22A/F) and designed assay-sets for 6A/C; 18B/C/F; 18C/F, 18F and 22F was validated through blind analysis of 1973 archived clinical samples collected from South African children ≤ 5-years-old (2009–2011), previously serotyped with the culture-based Quellung method. All assay-sets were efficient (92–101%), had low variation between replicates (R^2^ > 0.98), and were able to detect targets at a limit of detection (LOD) of < 100 Colony-Forming-Units (CFU)/mL of sample. There was high concordance (Kappa = 0.73–0.92); sensitivity (85–100%) and specificity (96–100%) for Fluidigm compared with Quellung for serotyping 6A; 6B; 6C; 18C and 22F. Fluidigm distinguishes vaccine-serotypes 6A, 6B, 18C, next-generation PCV-serotype 22F and non-vaccine-serotypes 6C, 6D, 18A, 18B, 18F and 22A. Discriminating single serotypes is important for assessing serotype replacement and the impact of PCVs on vaccine- and non-vaccine serotypes.

## Introduction

*Streptococcus pneumoniae* (pneumococcus) remains a leading cause of morbidity and mortality globally, causing an estimated 294 000 deaths (uncertainty range [UR] 192 000–366 000) in children 0–59 months without HIV in 2015^1^. Current PCV formulations include vaccine-serotypes (VT) 6A (PCV13), 6B and 18C (PCV13, PCV10). Serotype 22F is included in next-generation PCV15 and PCV20^2,3^. Before the introduction of PCVs, serogroup 6 was associated with 14–18% of invasive pneumococcal disease (IPD) episodes worldwide, whereas serotype 18C was the fifth most common serotype identified in IPD cases in developed nations^4,5^. Since the introduction of PCV, IPD due to serotypes 6A, 6B, and 18C have declined, however, 6A is still a common VT identified in carriage studies^6,7^. Serotype 22F has a high invasive disease potential and is increasing globally^8^.

The referent standard for pneumococcus detection and serotype identification has been the culture-based Quellung method which is not very sensitive and is costly^9,10^. Real-time PCR provides faster diagnosis and higher sensitivity^11^; however, due to genetic similarity some serotypes have been difficult to distinguish with PCR. Serogroups 6A/B; 6C/D; 18A/B/C and 22A/F were detected using real-time PCR^11^ and further distinguished with sequencing-based methods^12–14^ or conventional PCR combined with gel electrophoresis^15^. Sequential multiplex PCR has now been expanded to differentiate serogroups 6 and 22 but not 18^16^. Distinguishing serogroups 6, 18, and 22 into single serotypes entirely in a high-throughput comprehensive molecular reaction-set has not been described.

Serotypes within serogroups 6, 18 and 22 are antigenically, biochemically, and genetically distinct^13,17–21^. The genetic basis of capsule variation in 6A and 6C relative to 6B and 6D involves a single nucleotide polymorphism (SNP) in the rhamnosyl-transferase gene (wci*P*α in 6A/C, wci*P*β in 6B/D)^18,22,23^. Serotype 6B ‘sub-class II’ (6E) is a genetic variant expressing a 6B capsule that is neither biochemically nor antigenically distinct from 6B^24^. Serotypes 6F, 6G, and 6H express hybrid capsules, serologically and biochemically distinct within serogroup 6^25,26^. Serotype 18A does not contain the acetyl-transferase gene (wci*X*) present in 18B/C/F. Relative to 18C/F, serotype 18B contains a SNP in wci*X*. Serotype 18F contains an additional acetyl-transferase gene (wcx*M*), also present in 16F and 28AF^21^. Within serogroup 22 the genetic locus from wcw*A* (glycosyl-transferase gene) to wcw*C* (acetyl-transferase gene) is heterogeneous^13^.

In this study, nanofluidic real-time PCR (Fluidigm®) was used to distinguish individual serogroup 6 and serogroup 18 serotypes by including Dual Priming Oligonucleotide (DPO) forward primers to target the SNPs for 6A/C and 18C/F. Further, a thermodynamically modified Locked Nucleic Acid (LNA) probe was designed to detect 18C/F. Modified primer and probe strategies have not previously been utilised within the Fluidigm system.

## Results

### Performance of Fluidigm for identification of individual serotypes

**Figure 1:**
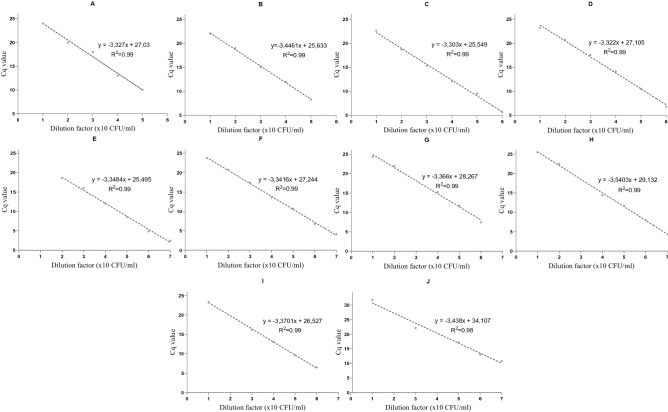
Standard curves constructed from duplicate serial dilutions to derive the linear equation, efficiency, and reproducibility for each serogroup 6, 18 and 22 assay-set. Panel A shows the culture standard for serotype 6B assessed with the assay-set to detect 6A/B/C/D; panel B is 6A assessed with the 6A/C assay-set; panel C is 6C assessed with the 6C/D assay-set; panel D is 6D with the 6C/D assay-set; panel E is 18C with the 18A/B/C/F assay-set; panel F is 18C with the 18B/C/F assay-set; panel G is 18C with the 18C/F assay-set; panel H is 18F with the 16F/18F/28A/F assay-set; panel I is 22A with the 22A/F assay-set and panel J is 22F with the 22F assay-set.

When assessed using the standard culture control strains, all assays and applied algorithms amplified their respective targets effectively. The efficiency (90–110%) and slope (− 3.6 ≥ slope ≥  − 3.3) of each of the serogroup 6, 18 and 22 assay-sets were within the prescribed ranges in amplifying control strains of known CFU/mL, with low variation between replicates (R^2^ > 0.98) (Table 1; Fig. 1).

**Table 1:** Performance of serogroup 6, 18 and 22 assay-sets in the Fluidigm.

Assay	Culture strain	LOD (CFU/mL)^†^	Linear equation	R^2^	Efficiency [− 1 + 10^(−1/m)^]
6A/B/C/D	6B	10^1^	− 3.327x + 30.357	0.99	100%
6A/C	6A	10^2^	− 3.4461x + 29.079	0.99	95%
6C/D	6C	10^2^	− 3.303x + 25.549	0.99	101%
6C/D	6D	10^1^	− 3.322x + 27.105	0.99	100%
18A/B/C	18C	10^2^	− 3.3484x + 22.148	0.99	99%
18B/C/F	18C	10^1^	− 3.3416x + 30.586	0.99	99%
18C/F	18C	10^2^	− 3.3743x + 31.732	0.99	98%
16F/18F/28A/F	18F	10^1^	− 3.5403x + 29.132	0.99	92%
22AF	22A	10^2^	− 3.3701x + 26.527	0.99	98%
22F	22F	10^2^	− 3.438x + 30.669	0.98	95%

^†^All LOD calculations were based on triplicate dilutions of g-Blocks, except for assays 6A/C and 18C/F where relevant culture controls were included.

The modified oligonucleotide strategies, including the DPO assays targeting 6A/C and 18C/F, combined with an LNA probe (18C/F), designed as part of this study were efficient (95% and 98% respectively), had low variation between replicates (R^2^ = 0.99), and were able to detect 6A/C and 18C/F at a LOD of 10^2^ CFU/mL (Table 1). Further, there was no cross-reactivity between serogroup 6 assay-sets (four serotypes) or serogroup 18 assay-sets (four serotypes) and the other 82 control strains for pneumococcal serotypes and eight nasopharyngeal colonisers included but not targeted by these assays.

### Detection of individual serotypes by Fluidigm® and culture

In the 1973 archived clinical samples, the serogroup 6 assay-sets and the applied algorithms were able to distinguish serogroup 6 to individual serotypes with 96–99% specificity, 85–100% sensitivity and excellent concordance compared to Quellung for 6A,6B and 6C (Kappa = 0.86, 0.73 and 0.91 respectively). Serotype 18C was distinguished within serogroup 18 with 99.9% specificity, and 88.9% sensitivity and with excellent concordance compared to uellung (Kappa = 0.86). Finally, serotype 22F was detected with 100% specificity, 85.7% sensitivity and excellent concordance (Kappa = 0.92) compared with Quellung. There was no significant difference between Quellung and Fluidigm for detection of individual serotypes 6A, 6C, 18C and 22F (McNemar’s test: p > 0.05). There was a significant difference in detection of serotype 6B (McNemar’s test: p < 0.01), where 6B was detected in 73 additional samples with Fluidigm. Fluidigm detected the same serotypes as Quellung for most of these samples (83%; n = 43/52) in addition to serotype 6B. In the remaining samples no pneumococcus was detected by Quellung (n = 12) or were allocated as 6A (n = 9) due to the bacterial load for these samples approaching the limit of detection and not detected by the assay-set targeted at 6A/C.

Specificity, sensitivity, and concordance could not be assessed for serotypes 6D, 18A, 18B, 18F and 22A as these are minor serotypes and no positive samples were detected by Quellung for comparison with Fluidigm.

## Discussion

The novel DPO, LNA, and other assay-sets designed here, combined with previously published serogroup 6, 18, and 22 assay-sets effectively discriminated between current PCV-formulation (PCV10, PCV13) VT (6A and 6B, 18C), next-generation PCV-formulation (PCV15, PCV20) VT 22F and NVT (6C, 6D, 18A, 18B, 18F and 22A) within a nanofluidic molecular serotyping reaction-set. Serogroups 6, 18, and 22 have previously been discriminated using conventional PCR^15,27,28^, or sequencing-based methods^12,13,29^. Sequencing-based methods are expensive, not all laboratories have access to sequencing platforms, bioinformatics software and expertise. Further, some methods were unable to fully distinguish 6C/D or 18B/C^29^. Microarray assays have been developed, but these are also costly and require initial time-consuming culture steps^30^. Recently the CDC sequential multiplex real-time serotyping PCR was updated with two and three additional assay-sets respectively to detect and discriminate serogroup 6 (6B/D, 6A/B) and 22 (22A/F, 22A, 22F) respectively^16^. The CDC’s unique 6B/D assay-sets utilized patented ‘BHQ-plus’ probes that include modified C and T nucleotides as duplex stabilizing chemistry to target the SNP that discriminates these serotypes. Our reaction-set was designed for comprehensive and high-throughput serotyping within a single nanofluidic real-time PCR, hence we used a compact strategy of assay-sets by designing just one additional assay-set each to further serotype serogroups 6 and 22. A benefit of our DPO primers is that they are not patented and may be purchased from any manufacturer globally to be incorporated with other probe chemistries if required.

No previous high-throughput real-time PCR assay has been reported to fully distinguish serogroup 6, 18, and 22^10,31–33^ within a single reaction-set or made use of modified primer or probe strategies such as LNA-probes or DPO primers as utilised in this study. This is the first study to validate the use of thermodynamically modified assay-sets within the Fluidigm platform.

Comprehensive accurate serotyping is essential where VTs may still be circulating and to assess the relative benefit of next-generation PCVs that will include serotype 22F. Current published molecular assays, including the serogroup 6 and 18 described here, now enable detection of PCV13-VT 1, 3, 4, 5, 6A, 6B, 14, 18C, 19A, 19F, and 23F individually^10,11^. Our reaction-set is the first to distinguish all PCV13-serotypes except serogroup 9A/V and 7A/F to single serotypes. Relative to 9 V, 9A has a frameshift deletion in a G-polymer region surrounded by an AT-rich region^21^. Similarly, 7A lacks a side chain due to a frameshift mutation in the glycosyl transferase (wcw*D*) gene^21^ located in a poly-T region within a GC region. These molecular features make the SNPs in 9 V and 7F difficult to target and thermodynamically improbable to distinguish with molecular methods other than sequencing-based approaches.

The performance (sensitivity, specificity, and limit of detection) of the serogroup 6, 18, and 22 reaction-sets in the Fluidigm platform is comparable with other high-throughput strategies including the TaqMan Array Cards and the same Fluidigm platform including using different real-time PCR chemistry^10,33,34^. The performance of this reaction-set demonstrates that modified primers (DPO) or probes (LNA) are easily adapted to high-throughput real-time PCR, including where specific target pre-amplification is undertaken in multiplex (up to 34 assay-sets per tube). The successful combination of a DPO primer with a LNA probe to target 18C/F is a novel strategy. There is capacity for 96 samples and controls to be tested against 96 targets within a single run for the Fluidigm 96.96 Dynamic Array IFC (integrated fluidic circuit) that we utilized in the study, however the reaction-sets described here can be easily adapted to other real-time PCR platforms for lower through-put applications.

This study was limited in that non-pneumococcal *Streptococcus* species were not included in the validation of the assay-sets. Previous studies have validated the assay-sets for 6A/B/C/D, 6C/D, 18A/B/C, and 22AF against non-pneumococcal *Streptococcus*^10^ and our algorithm included that samples must be detected with the serogroup 6 assay-set (6A/B/C/D), 18A/B/C, and 22AF to be assigned a serogroup 6, 18 or 22 type respectively, hence the designed assays are unlikely to detect other non-pneumococcal species. The methods described here are not currently aimed at detecting the hybrid serotypes 6F/G/H, as no reference specimens or clinical samples were available to validate the detection of these additional serotypes. Serotype 6B ‘sub-class II’ (6E) is a genetic variant of 6B, and the genetic regions targeted here are ubiquitous in 6B sub-classes so would be correctly assigned serotype 6B^24^. Prospective studies should include surveillance for hybrid serotypes and conduct confirmatory sequencing for isolates typed as serogroup 6 where PCVs are in use and residual serogroup 6 carriage is observed.

The Fluidigm could not be fully evaluated against the Quellung-method for detection of serotypes 6D, 18A/B/F or 22A in clinical isolates as these serotypes were not detected in the archived clinical samples by Quellung, most likely due to their low prevalence in Africa. Nevertheless, our algorithm correctly identified cultured control strains and would be able to correctly discern serotype 6D, 18A, 18F, 22A and 22F in clinical isolates. While the culture-based Quellung method is sensitive and specific, PCR is more sensitive and capable of detecting multiple co-colonising or culture non-typeable serotypes, even at a low density^35,36^. Genetic heterogeneity or the presence of a target of interest detected with molecular methods are not always accurate predictors of phenotypic capsule type and capsular expression in isolates^24^. Since the positive predictive value of Quellung is not perfect in cases of multiple serotype carriage or at low density, discrepant serotype positive designations by PCR are resolved as putative by confirming the presence of pneumococcal reference genes^37^. As Fluidigm PCR is compared to Quellung in this study, this may affect the measured concordance, for example serotype 6B where this serotype was detected in an additional 73 samples with Fluidigm. The benefit of real-time PCR is the enhanced detection of multiple serotypes carried concurrently compared with the Quellung method. Indeed, the serotypes assigned by the Quellung method were detected correctly in addition to serotype 6B in samples with multiple colonizing serotypes detected by our Fluidigm method. Further, the clinical samples that were re-analysed with Fluidigm as part of this study, had undergone multiple freeze–thaw cycles. This may have affected the sensitivity of Fluidigm compared with Quellung.

The DPO assay-sets targeting wci*P*α (6A/C) and wci*X* (18C/F) in conjunction with the designed wci*X* (18B/C/F), wcx*M* (18F), wcw*A* (22F), and published serogroup 6, 18 and 22 (6A/B/C/D; 18A/B/C; 22AF)^11^ and 6C/D^38^ assay-sets using the applied algorithms, can be used to correctly serotype circulating serogroup 6, 18 and 22 to individual serotypes using Fluidigm® real-time PCR in clinical samples.

## Methods

DPO strategies overcome the limitations of primer design by combining the thermodynamic advantages of two isolated priming regions to enable sensitive and specific detection of SNPs in low GC regions^39,40^. LNA-based probes contain conformation-modified bases with a methylene bridge connecting the 2′-oxygen and 4′-carbon of the ribose ring in a nucleic acid base thereby increasing the melting temperature (Tm) for sensitive detection of low GC target regions^41^.

### Primer design

We included oligonucleotide primers and dye-labelled Minor Grove Binding (MGB) probes targeting serogroup 6 (6A/B/C/D), sub-group 6C/D, subgroup 18A/B/C, and serogroup 22 (22A/F) from published sequences^11,38^ that have been optimized previously on the Biomark™ HD platform real-time PCR (Fluidigm)^33^ in the reaction-set.

Genbank FASTA sequences of representative serogroup 6, 18, and 22F capsular genes (Table 2) were aligned separately for each serogroup in BioEdit using ClustalW Multiple alignment^42,43^. Standard primers and dye-labelled MGB probes were manually designed to detect the genes for wci*X* in 18B/C/F, wcx*M* in 18F, and wcw*A* unique to 22F, based on the Genbank accession numbers in Table 2. Sequences for serotypes 6B ‘sub-class II’ (6E), 6F, 6G, and 6H were included only for in silico analysis of their detection pattern. All oligonucleotide sequences that are listed in Table 2 were analysed to ensure specificity in silico using the Basic Local Alignment Search Tool (BLAST; http://www.ncbi.nlm.nih.gov/BLAST/) prior to inclusion.

**Table 2:** Serotype-specific capsular sequences used to design the Dual Priming Oligonucleotide primers (DPO); Locked Nucleic Acid (LNA) probes and standard assays.

Serotype	Genbank accession number:	References
6A	CR931638	Bentley et al.^44^
	JF911487–JF911497	Elberse et al.^45^
6B	CR931639	Bentley et al.^44^
	JF911498–JF911508	Elberse et al.^45^
	KT907353^†^; KU168827	Burton et al.^24^
6C	EF538714	Park et al.^18^
	HQ662201; HQ662202	Song et al.^20^
	JF911509; JF911510; JF911515	Elberse et al.^45^
6D	FJ899602	Jin et al.^46^
	HV580364*	Kapatai et al.^13^
	HQ662205; HQ662209; HQ662210; HQ662216; HQ662217	Song et al.^20^
6″E”	LT594599^†^	Kapatai et al.^13^
6F	KC832410	Oliver et al.^25^
6G	KC832411	Oliver et al.^25^
6H	KJ874439	Park et al.^26^
18A	CR931671	Bentley et al.^44^
18B	CR931672	Bentley et al.^44^
18C	CR931673	Bentley et al.^44^
18F	CR931674	Bentley et al.^44^
22A	CR931681	Bentley et al.^44^
22F	LT594600	Kapatai et al.^13^

^†^Serotype 6B ‘sub-class II’ or 6E; *Sequenced by Park, I. and Nahm, M.H.

**Figure 2:**
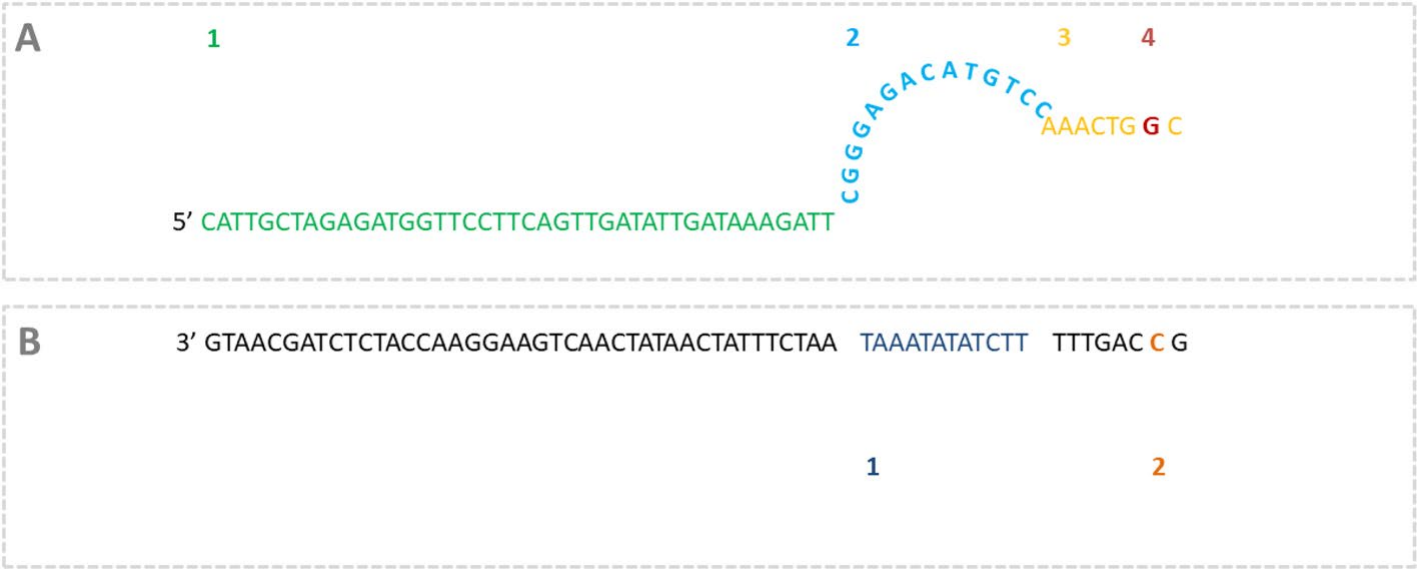
Schematic representation of the Dual Priming Oligonucleotide (DPO) forward primer (**A**) for the detection of wciPα and the target 6A/C sequence (**B**) as an example of the DPO designs used in this study. DPO primers combine the thermodynamic advantages of two priming regions, a short highly specific ‘foot’ sequence terminating at the 3’ end, and a longer stable and sensitive 5’ ‘anchor’ sequence with their action separated by a non-complimentary ‘bridge’ sequence that forms a bubble. The ‘bridge’ keeps the highly specific foot sequence close to the target sequence by connecting it to the anchor, increasing the frequency of target-primer hybrid formation to enable highly specific primer extension. Due to the dual priming nature of these oligonucleotides, the extension will not proceed if the 3’ short foot sequence is mismatched. A1 shows the ‘anchor’ sequence which is the first priming region, A2 is the ‘bridge’ sequence which is non-complimentary to the intervening sequence (B1) which is not a priming region and the second priming region with the ‘foot’ sequence (A3) containing the interrogating nucleotide (A4) which targets the SNP on the wciPα target strand (B2).

To further distinguish individual serogroup 6 and 18 serotypes, DPO primers and a LNA probe were newly designed (Table 3). The forward DPO for serogroup 6A/C and 18C/F were designed based on the Genbank sequence accession numbers in Table 2. The highly specific and shorter 3’ ‘foot’ sequence to target the SNPs in 6A/C and 18C/F were designed based on the capsule gene locus for wci*P*α, and wci*X*_CF_ respectively with the ‘interrogating nucleotide’ (to detect the target SNP) located at the penultimate 3’ position. The location of the longer and thermodynamically stable 5’ ‘anchor’ sequence was predicated on the length of the non-complimentary ‘bridge’ sequence (that separates the two priming regions) and the location of the upstream SNP specific ‘foot’ sequence (example for 6A/C in Fig. 2). A standard tagged Integrated DNA Technologies (IDT) PrimeTime® fluorescent probe (6A/C) and a PrimeTime LNA® real-time PCR probe (18C/F) and reverse primers were designed manually for both DPOs.

**Table 3:** Primer and probe assay-sets for serogroup 6, 18 and 22 detection and serotype discrimination.

Oligonucleotide	Sequence 5′–3′	Target	References
6A/B/C/D Forward	AAGTTTGCACTAGAGTATGGGAAGGT	wci*P*	Azzari et al.^11^
6A/B/C/D Reverse	CTTGTATCGAAGACAYGGACATAATGT		
6A/B/C/D Probe^†^	TGTTCTGCCCTGAGCAACTGG		
6A/C-DPO Forward	CATTGCTAGAGATGGTTCCTTCAGTTGATATTGATAAAGATTCGGGAGACATGTCCAAACTGGC	wci*P*_α_	This study
6A/C Reverse	CGATACAAGACCAGTTGC		
6A/C Probe^‡^	TTTGCACTAGAGTATGG		
6C/D Forward	TTGGGATGATTGGTCGTATTAG	wci*N*_β_	Azzari et al.^11^
6C/D Reverse	CGAACTGAAGAACTAATTGAAGAG		
6C/D Probe^†^	CCACGCAATTCGCCATC		
18A/B/C Forward	CCTGTTGTTATTCACGCCTTACG	wci*W*	Azzari et al.^11^
18A/B/C Reverse	TTGCACTTCTCGAATAGCCTTACTC		
18A/B/C Probe^†^	AACCGTTGGCCCTTGTGGTGGA		
18B/C/F Forward	CAGGATTTCTAACTCTGATTGAA	wci*X*_BCF_	This study
18B/C/F Reverse	AGCAAAATCTAACGTCCAGAG		
18B/C/F Probe^†^	CTTGTATGCTTATGGTCTTTTCGATTA		
18C/F-DPO Forward	CCAAATTGGAGTGTTTTACAAAGTATTAGCTCGATTTGCTGTACACGTCGACGCTTCAATTTCAGG	wci*X*_CF_	This study
18C/F Reverse	TCTTTCAAATACAACTCTTAGATTTCCTTGTG		
18C/F LNA-Probe^‡^	TGagtTTATTGATAATttcC		
16F/18F/28AF Forward	TGGTTTCGGACTCTTTCGTGG	wcx*M*	This study
16F/18F/28AF Reverse	CTAAGATAGAAACTCCTTGTCCAATG		
16F/18F/28AF Probe^†^	GGTTGTACGTGGAATCGGATTTGGTC		
22AF Forward	TCTATTAAATAACCCATTGGAATTGAAACG	wcw*V*_A_	Azzari et al.^11^
22AF Reverse	TCGCAATTGAAGACCACATAAACTG		
22AF Probe^†^	TCCGTAATTCGCTTATGGGCACATTCTCCA		
22F Forward	GAAGATTGTCCACCTTATATCC	wcw*A*_F_	This study
22F Reverse	TCGGCACAATCAAAATATC		
22F Probe^†^	CGGTTATTTCACAAAAGACACGGTTGG		
LytA Forward	TCTTACGCAATCTAGCAGATGAAGC	LytA	Carvalho et al.^47^
LytA Reverse	GCACGAATAACCAACCAAACAAC		
LytA Probe^†^	CCGCAACTCATCAAGGATTTCTGTTACCA		
PiaB Forward	CATTGGTGGCTTAGTAAGTGCAA	PiaB	Brown et al.^48^
PiaB Reverse	TACTAACACAAGTTCCTGATAAGGCAAGT		
PiaB Probe^†^	TGTAAGCGGAAAAGCAGGCCTTACCC		

Nucleotides in lower case correspond to locked nucleic acid (LNA) modified bases.

^†^5′-FAM- NFQ-3′ labelled dye (Thermofisher).

^‡^5′6-FAM- BHQ1-3′ labelled dye (Integrated DNA Technologies).

The final reaction-set included primer–probe sets which amplified target regions of serogroup 6 types with wci*P* (6A/B/C/D), wci*N*β (6C/D), wci*P*α (6A/C); serogroup 18 with wci*W* (18A/B/C), wci*X* (18B/C/F), wci*X* (18C/F), wcx*M* (18F) and serogroup 22 with wcw*V* (22A/F) and wcw*A* (22F) (Table 3).

Since each serotype was not targeted individually, an algorithm combining results from published primer–probe sets (6A/B/C/D, 6C/D, 18A/B/C, and 22A/F) and newly designed sets (6A/C, 18BCF, 18C/F, 18F and 22F) was applied to distinguish individual serogroup 6, 18 and 22 serotypes (Table 4 and further described in supplementary).

**Table 4:** Algorithm to discriminate serogroups 6, 18 and 22 to individual serotypes using the described reaction sets.

Serotype	6A	6B	6C	6D	6F^†^	6G^†^	6H^†^	18A	18B	18C	18F	22A	22F
6A/B/C/D	+	+	+	+	+	+	+	−	−	−	−	−	−
6A/C	+	−	+	−	+	−	+	−	−	−	−	−	−
6C/D	−	−	+	+	−	−	−	−	−	−	−	−	−
18A/B/C	−	−	−	−	−	−	−	+	+	+	−	−	−
18BCF	−	−	−	−	−	−	−	−	+	+	+	−	−
18CF	−	−	−	−	−	−	−	−	−	+	+	−	−
16F/18F/28AF	−	−	−	−	−	−	−	−	−	−	+	−	−
22AF	−	−	−	−	−	−	−	−	−	−	−	+	+
22F	−	−	−	−	−	−	−	−	−	−	−	−	+

^†^Theoretical detection pattern predicted in silico, no bacterial isolates or clinical isolates were available to confirm.

To confirm the presence of *S. pneumoniae*, pan-pneumococcal reference genes *LytA* (pneumococcal autolysin gene)^47^ and PiaB (permease gene of the pia ABC transporter)^48^ were also included in the reaction-set. Samples in which a serotype-specific target gene was detected, also needed to be positive for the two pneumococcal reference genes to be confirmed as *S. pneumoniae*.

### Bacterial and pneumococcal reference isolates

Control strains for *S. pneumoniae* serotypes (1; 2; 3; 4; 5; 6A; 6B; 6C; 6D; 7A; 7B; 7F; 7C; 8; 9A; 9L; 9 N; 9 V; 10A; 10B; 10C; 11A; 11B; 11C; 11D; 11F; 12B; 12F; 13; 14; 15A; 15B; 15C; 15F; 16A; 16F; 17A; 17F; 18A; 18B; 18C; 18F; 19A; 19B; 19C; 19F; 20; 21; 22A; 22F; 23A; 23B; 23F; 24A; 24B; 24F; 25A; 25F; 27; 28A; 28F; 29; 31; 32A; 32F; 33A; 33B; 33C; 33D; 33F; 34; 35A; 35C; 35F; 35B; 36; 37; 38; 39; 40; 41A; 42; 43; 44; 45; 46; 47A; 47F; 48) and other nasopharyngeal species (*Acinetobacter baumannii*, *Haemophilus influenzae*, *Klebsiella pneumoniae*, *Moraxella catarrhalis*, *Neisseria lactamica*, *Neisseria meningitidis*, *Staphylococcus aureus* and *Streptococcus pyogenes*) were obtained from the National Institute for Communicable Diseases, South Africa, Murdoch Children’s Research Institute, Australia and Vaccines and Infectious Diseases Analytics Research Unit, Soweto, South Africa. DNA from these strains were used to optimize the PCR assay-sets.

### Bacterial culture and DNA extraction

Control isolates were grown according to standard microtiter culture methods to quantify their relative density (colony forming units, CFU/mL) for use as reference standards and quantification calibrators. The density of viable cells (CFU/mL) was calculated using a serial dilution method (further detailed in Supplementary). Total DNA was extracted from 1 ml THB or BHI into 100 μL of elution buffer using the BioMérieux NucliSens easyMAG® automated benchtop nucleic acid extraction system (BioMérieux, Marcy l′Etoile, France) with standard reagents and protocols. Extracted DNA from reference strains were stored at – 20 °C until assayed. Where required, bacterial isolates of known CFU/mL were used as positive template controls, in addition to gBlocks™ synthetic external calibrators (Supplementary Tables S1 and S2).

### Clinical samples

Two cross-sectional surveys were undertaken in Agincourt (May–October 2009) and Soweto (May 2010–February 2011) to assess the prevalence of serotype-specific pneumococcal colonisation during the early introduction of PCV7 in South Africa^49,50^. Nasopharyngeal swabs (NPS) in STGG (Skim-milk-tryptone-glucose-glycerine transport media)^51^ were collected from households (Agincourt) and children 0–12 years old (Soweto) and serotyped using the Quellung method, as previously described^49,50^.

In this study, NPS in STGG from children aged 0 to 5-years-old were re-tested by Fluidigm to validate the assay-set for accurate serotyping in clinical samples compared with the Quellung method. Serotype data for 1973 samples were therefore available for comparison between Fluidigm PCR and Quellung method. Nucleic acids were extracted from 400 µL of STGG and eluted into 100 µL of elution buffer using the BioMérieux NucliSens easyMAG® according to standard manufacturer protocols. No-template-control (NTC) that was STGG alone was included in each batch of clinical samples (n = 23) that were extracted. Nucleic acids were stored at – 20 °C until assayed.

### Fluidigm Real-time qPCR

**Figure 3:**
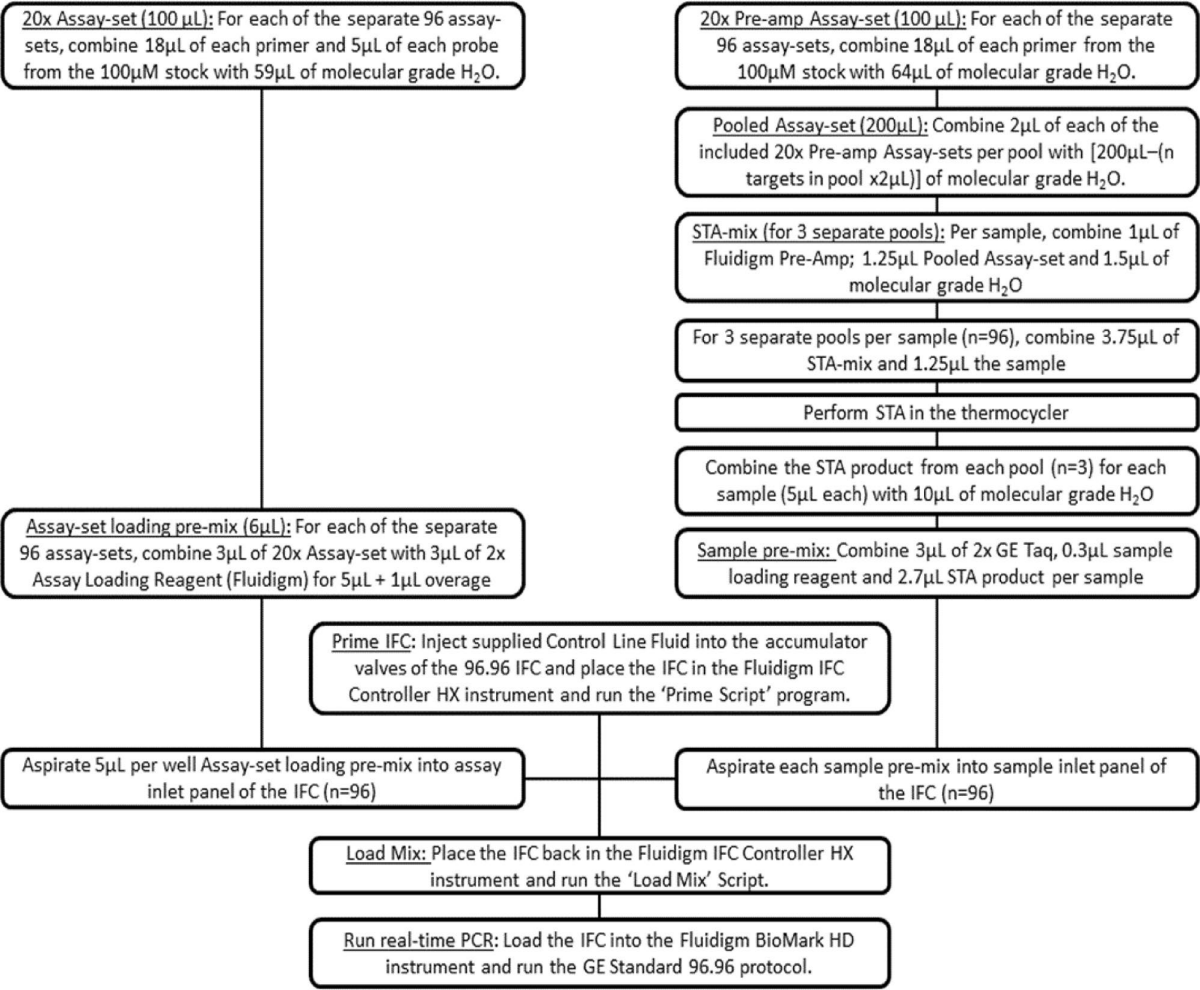
Flow diagram of the Specific Target Amplification (STA) within single 0.6 mL PCR tubes in the Bio-Rad T100 Thermal Cycler and the nano-fluidic high throughput real-time PCR within the 96.96 Gene Expression (GE) Dynamic Array Integrated Fluidic Circuit (IFC) carried out in the IFC Controller HX and the BioMark HD (Fluidigm).

The serogroup 6, serogroup 18 and serogroup 22 assay-sets designed in this study were included in a high-throughput nanofluidic real-time PCR serotyping panel on the Fluidigm platform that was optimised as described previously^33^ and here was expanded to include other published assay-sets to increase the number of individual serotypes detected. The Fluidigm workflow is summarized in Fig. 3. Primers for all assay-sets were separated into three specific target amplification (STA) multiplex pools (Table S3) to prevent any cross-reactivity of primers.

### Specific target amplification/pre-amplification pools

The process of preparing each STA mix is detailed in Fig. 3 and further described in supplementary. For each pool, each included assay-set (n = 30–32 assay-sets per pool; Table S1) were combined to make up 200 µL of a multiplex STA pool. The final STA mix (5 µL) per sample contained 1.25 µL pooled assays at a final concentration of 45 nM for each primer, 1 µL Fluidigm Gene Expression (GE) Pre-Amp reagent, 1.50 µL ddH_2_O and 1.25 µL of sample. Reactions were amplified in a T100 Thermal Cycler (Bio-Rad, Inc, CA, USA) and cycling conditions were: 95 °C for 2 min, 14 cycles of 95 °C for 15 s and 60 °C for 4 min, and a hold of 4 °C. STA products (5 µL) from the three PCR reactions were then combined and diluted with 10 µL H_2_O to further dilute any remaining primers to less than 9 nM each in a final volume of 25 µL and tested in the Fluidigm.

The Fluidigm 96.96 GE Dynamic Array IFC™ was primed and loaded according to manufacturer specifications and placed in the Biomark™ HD for Fluidigm PCR, using the following thermal cycling conditions: 50 °C for 2 min, 70 °C for 30 min, 25 °C for 10 min, 50 °C for 2 min, 96.5 °C for 10 min followed by 40 cycles of 96 °C for 15 s, 60 °C for 60 s. Data were analysed with the supplied Real-Time PCR Analysis Software for the BioMark™ HD instrument (Fluidigm® Corporation, CA, USA) using manually defined thresholds and a Cycle of quantification (Cq) cut off value of 35.

### Primer optimization

Secondary structures, self-annealing sites, and melting temperature (Tm) of each assay-set were predicted using the online tool OligoCalc^52^. Duplicate serial dilutions of total DNA from culture controls with CFU/mL of 10^0^ to 10^6^ were used to develop standard curves for all assay-sets to assess efficiency and reproducibility (R^2^). Triplicate dilutions at 10^0^ to 10^3^ CFU/mL or copy number equivalents (for Synthetic gBlock external calibrators) were included to assess the limit of detection (LOD), that was defined as the lowest concentration that was detected in triplicate. Further, all assays were tested for cross-reactivity against 90 pneumococcal positive culture controls, including individual serotype 6,18, 22 serotypes and 8 other common nasopharyngeal bacterial colonisers.

### Assay-set validation for use with clinical samples

To validate the serogroup 6,18 and 22 serotyping reaction-sets for use in clinical isolates, a blind analysis was conducted on 1973 clinical samples described above. Synthetic gBlock external calibrators (positive controls), and no-template controls (NTC) were included within each Fluidigm assay.

### Statistical analysis

The slope (m) of the linear equation (y = mx + c) generated from the standard curves of tenfold serial dilutions of calibrators (bacterial culture controls or gBlock) were used to calculate the percent efficiency (E) for each assay-set (E = [10^(−1/m)−1^] × 100). Analyses were done in Stata, version 13.0, including applying relevant algorithms for serotype assignment. The McNemar’s test was used to determine the sensitivity of the Fluidigm® assay-sets for serotyping serogroups 6, 18 and 22 to individual types compared to the gold standard culture-based Quellung. Cohen’s kappa coefficient was used to concordance for each of the archived clinical samples serotyped using Fluidigm and the described algorithm compared with the Quellung results. Results were considered significant when *p-*values were ≤ 0.05.

### Ethial approval

Ethical consent was obtained from the Medical Human Research Ethics Committee (HREC) of the University of Witwatersrand for the initial collection of the included samples (Soweto Cohort HREC: M090115; Agincourt Cohort HREC: M090114). Informed consent was obtained from all participants and their legal guardians as part of the original study in which the samples were obtained. All experimental protocols were approved by Medical Human Research Ethics Committee (HREC) of the University of Witwatersrand (HREC: M170314). All methods were performed in accordance with the relevant local and international guidelines and regulations for Good Clinical Practice.

## Supplementary Information

Supplementary Information.

## Data Availability

The datasets generated during and/or analysed during the current study are available from the corresponding authors on reasonable request.
