# Supplementary material for: High-throughput nanofluidic real-time PCR to discriminate Pneumococcal Conjugate Vaccine (PCV)-associated serogroups 6, 18, and 22 to serotypes using modified oligonucleotides
Source: Sci Rep. 2021 Dec 9;11:23728. doi: 10.1038/s41598-021-03127-9 (PMC8660885; doi:10.1038/s41598-021-03127-9)
Supplement: Supplementary file 1 — Supplementary Information. [file 41598_2021_3127_MOESM1_ESM.pdf]

High-throughput nanofluidic real-time PCR to discriminate Pneumococcal Conjugate Vaccine (PCV)-associated serogroups 6, 18, and 22 to serotypes using modified oligonucleotides.

SL Downs[1, 2]*, SA Madhi[1, 2], L Van der Merwe[1, 2], MC Nunes[1, 2] & CP Olwagen[1, 2]*.

Supplementary information:

Interpreting the Algorithm for serotyping serogroup 6, 18 and 22.

Samples/isolates detected with the ubiquitous serogroup 6 wci*P* (6A/B/C/D) assay-set and our newly designed 6A/C (wci*P*α) DPO assay-set, but not detected by the 6C/D (wci*N*β) assay-set were serotyped as 6A. Isolates that were detected with the same assay-sets (6A/B/C/D and 6A/C) but also detected with the 6C/D (wci*N*β) assay-set were serotyped as 6C. Isolates detected with 6A/B/C/D and 6C/D but not with the assay set for 6A/C were serotyped as 6D. Finally, isolates detected with 6A/B/C/D but not detected with the assay-sets for 6A/C or 6C/D were serotyped as 6B. Similarly, serogroup 18 was serotyped using the published primer-probe set targeting 18A/B/C and the designed sets targeting 18B/C/F, 16F/18F/28AF and the DPO targeting 18C/F. Isolates detected with the wci*W* targeted assay-set for 18A/B/C but not detected with other serogroup 18 assay-sets were designated as serotype 18A. Isolates detected with the 18A/B/C assay-set and the assay-set targeted at wci*X* (18B/C/F) but not detected with other assay-sets were serotyped as 18B. Isolates detected with the 18A/B/C assay-set, the 18B/C/F assay-set and the DPO assay-set targeted at wci*X* contained in 18C/F but not detected with the assay-sets for wcx*M* (16F/18F/28AF) were serotyped as 18C. Any isolates detected by all serogroup 18 assay-sets excluding 18A/B/C were serotyped as 18F. Isolates detected by 22A/F but not 22F were designated as 22A. Isolates that were detected by both serogroup 22 assay sets (22A/F and 22F) were designated 22F. The interpretation of the algorithm is summarised in the manuscript (Table 4).

Bacterial culture and DNA extraction

Control isolates were grown according to standard microtiter culture methods to quantify their relative density (colony forming units, CFU/ml) for use as reference standards and quantification calibrators. Specifically, *S. pneumoniae* serotypes, *A. baumannii*, *K. pneumoniae*, *M. catarrhalis*, *N. lactamica*, *N. meningitidis*, *S. aureus* and *S pyogenes* control strains were streaked on 5% blood agar plates; *H. influenzae* strains were streaked onto chocolate agar plates for single colonies (using a four-way streak) and incubated in 5% CO_2 at 37°C for 12 hours. Single colonies (n=4-6) were picked and inoculated into Todd Hewitt Broth (THB) supplemented with 5% yeast (*S. pneumoniae*); THB (*A. baumannii*, *K. pneumoniae*, *M. catarrhalis*, *N. lactamica*, *N. meningitidis*, *S. aureus* and *S pyogenes*) and brain heart infusion (BHI) for *H. influenzae* strains and grown in CO_2 at 37°C and 5% CO_2 to an OD_{260} of 0.1 (*S. pneumoniae*) or 1.0 (all other bacterial strains) to obtain an optimum concentration of 1 x 10^7 CFUs/ml.

The density of viable cells (CFU/ml) was calculated using a serial dilution method. A total of 200µl of THB or BHI was aspirated into a microtiter plate and a 1:10 serial dilution was performed with sterile Phosphate-buffered saline (PBS). The lowest 6 dilutions were plated as triplicate 20µl drops on the relevant 5% blood agar plates or 'chocolate' agar plates and individual colonies in each triplicate drop were counted following 20h of growth at 37°C and 5% CO_2.

The lowest concentration containing 5-50 colonies was used to estimate the density using the formula:

$$Density\ (CFU/ml) = CFU\frac{Drop\ 1,\ \ Drop\ 2,\ \ Drop\ 3}{3} * 50 * dilution\ factor$$

Total DNA was extracted from 1ml THB or BHI into 100µl of elution buffer using the BioMérieux NucliSens easyMAG® automated benchtop nucleic acid extraction system (BioMérieux, Marcy l'Etoile, France) with standard reagents and protocols. Extracted DNA from reference strains were stored at -20ºC until assayed. Where required, bacterial isolates were used as calibrated positive template controls, in addition to synthetic external calibrators (gBlocks™).

Synthetic external calibrators

Synthetic double-stranded template gene fragments (gBlocks) were designed based on the sequences for each serotype or bacterial target according to the pool in which the respective assay-sets were included. For each pool, three gBlocks were designed including nine to thirteen target regions. Target sequences were aligned in BioEdit, and the primer and probe sequences were aligned to the target sequence for each target assay-set. The intervening base pairs were removed within the target sequence, leaving the primer and probe binding regions of the target for a shortened sequence. Buffer sequences of random base pairs non-complimentary to any of the assay-sets or their targets are inserted on the 5' and 3' end (around 20-50bp) of the oligonucleotide and in between each target (around 25bp and primer or probe sequence (around 5-10bp). The combined buffer and target sequences were analysed in the online IDT gBlocks Gene Fragments Design Tool to check for any secondary strugtures. Target sequences within gBlocks are easily quantified as there is an equimolar ratio of all templates. The gBlocks properties and sequences used in this study are included in Table S1 and S2.

Specific Target Amplification (STA) Pools

Primer stocks were made up to 100µM concentration. Working stock assay-sets (primers) were made up to 18µM primer concentration. For each pool, 2µL of working stock for each included assay-set (n=30-32 assay-sets per pool; Table S3) were combined with molecular grade double-distilled H_2O (200µL– [No. assay-sets x 2µL]) to make up a 200µL of a multiplex STA pool. The final STA mix (5µL) per sample contained 1.25µL pooled assays at a final concentration of 45nM for each primer, 1µL Fluidigm® Gene Expression (GE) Pre-Amp reagent, 1.50µL ddH_2O and 1.25µL of sample.

<u>Fluidigm BioMark HD loading, sample, and assay preparation</u>

Control line fluid (Fluidigm) was added into the accumulator and the Fluidigm IFC was primed in the Fluidigm IFC Controller HX prior to loading. Once primed, a total of 5µL assay premix (2.5µL assay; 2.5µL loading reagent) for each target was aspirated into 96 assay inlets (final concentration: 9µM primers and 2.5µM probe). Pooled STA product (2.25µL) for each of the 96 samples including relevant controls were combined with 2.75µL sample premixes (2.50µL GE Taq, 0.25µL sample loading reagent) in separate tubes and aspirated (5µL) into sample inlets. The Fluidigm IFC Controller HX was used to load the samples and assays into the chip. The loaded chip was then placed in the BiomarkHD for Fluidigm PCR, using the following thermal cycling conditions: 50°C for 2 min, 70°C for 30 min, 25°C for 10 min, 50°C for 2 min, 96.5°C for 10 min followed by 40 cycles of 96°C for 15 sec, 60°C for 60 sec.

Table S1: External synthetic calibrator (gBlock) properties.

gBlock™ name	Assay-Set Targets †	Length (bp)	Amount (ng/µl) ‡	Gene equivalents (Copy number)
A1: Pool A gBlock, no. 1	1, 4, 5, 6A/B/C/D, 33D, LytA, 19F (Atypical), 7A/F, 8, 9A/L/N/V	1088	8,09	$1,36 \times 10^{10}$
A2: Pool A gBlock, no. 2	10C/F, 11A/D, 14, 20, Hib, 16F, 22F, 15B/C, 18A/B/C, 19B/F	1147	6,73	$1,07 \times 10^{10}$
A3: Pool A gBlock, no. 3	11F, 24A, 28A/F, 17A, 35F/47F, 41F, 12A/B/F/44/46, 23A/B/F, Eco, Sag, hIS1001, Nme	1225	8,53	$1,27 \times 10^{10}$
B1: Pool B gBlock, no. 1	3, 6A/C, 7B/C/40, 9A/V, 10B, 11A/B/C/E/F, 12B, 13, 15A/B/C/F, 16A, 34/37/17A, 18B/C/F, 19C	1474	3,82	$4,73 \times 10^{9}$
B2: Pool B gBlock, no. 2	19F, 22A/F, 23F, 25A/F, 33C, 35B, 46, 47A/F, Xis	1023	7,71	$1,38 \times 10^{10}$
B3: Pool B gBlock, no. 3	BexA, Nla, Mca, Spy, Sau, 16S, PiaB, PtxS1, plS1001, IS481, Sor	1122	5,13	$8,34 \times 10^{9}$
C1: Pool C gBlock, no. 1	15A/F, Ply, 11B/C, 16F/18F/28A/F, 17F, 19A, 21, 23B, 24B/F, 25A/F/38, 27, 29	1284	7,04	$1,00 \times 10^{10}$
C2: Pool C gBlock no. 2	31, 32A/F, 33A/F/37, 33B, 34, 35A/C/42, 45, 48, 2, 10A, 9L/N	1252	5,28	$7,70 \times 10^{9}$
C3: Pool C gBlock no 3	Aba, Kpn, 23A, BexB, IgA1, Pji, 36, 39, 41A, 43, 6C/D.	1160	4,11	$6,47 \times 10^{9}$

† Listed in order of position on gBlock; ‡ Average value of 3 successive Q-bit measurements

Table S2: External synthetic calibrator (gBlock) sequences

gBlock name	gBlock sequence
A1	TACCAGACGATGCTTATCAATGACCCCTATATGTGTGATATCGTGCGGTAATTGAAGCTATGAGTTGTGCTTGCCCTTGTATAGGGTCTCCAGAGTTGCTGGGGCCACATCACCGCAGGTATCCCGTCGCCGGTTGGGATGACATTTCTACGCACTATCTTTCCTATTGGATGGTTAGTTGGTGATA<br>GTTGATAAAGCATCAGCGACGGGGGTTGCTCTGGGTGCCATGAACTATTACGGGAGTATCTTATGTCTTTAATGGTTTGTCTCAGCAACTCTATTTGGCTGTGGGAATTCTAGTTTTAGGCTACTGGAATGCTGGCAGCGACGACCACCACACACACATGCAAGTTTGCACTAGAGTATGGGAAG<br>GTGTTGTTCTGCCCTGAGCAACTGGTGCTACTTGTATCGAAGACATGGACATAATGTTAACCTATGAACTCGATCAGAGAACGTATAGTCTTGCGACATTTCATCCACAACTAGTTTTTTATCAAAAAGACCTTGGCGAGGTGAGGTAACGCATGTGGAACATACCAAGCATCTCCATCTAAGTTC<br>TTACGCAATCTAGCAGATGAAGCTATTTGCCGAAAACGCTTGATACAGGGTATGCACGAATAACCAACCAAACAACTATTAATATGGTATATTTATGTCGCGTCCTTAGTTCTGGTTATTCGGGAATCCAGTTATGAAGGTGAGCTAACAGTGCGACACTTCGATTCGGTTCCTCATCCGCCCTAC<br>CGACAGTGGTACTAGAGGCGATGGCATGTGGCAAACCATAGTTGGCTATCGGCATGGTGGTGTTGTGAAATGATTAAGGAGGGCAAAAACGGTCTCCTAGCCACTCCAGCTATTCCCACTCATCAGTTTCCCATATGTTTCATTCTGATGGCAGATGGGTTGGGACGAGTAACGTTCGCTTCTTC<br>AATTATTGATTGCCCACGCAATGGGGATGCTGGAATGGGCAAAGGGTAGTAAGTTAATCATGCTAACGGCTCATCGACGAGAAAATCTTGGGGAACCGATGGAGAGCCCGAGTTAAGGTGGAGCTCAAGTAAATGGTCTACCGACACTACTCTCAGAAG
A2	TTCCACGGCTCAAGTAAATGGTCTTGATTAGTAAGGCAGGATGCATAAACCAGGAGGTACCGAGTTATGGATGTTCTTATTGGCTTGTGGAACAATATTCACAGTCTTGCCCTGTTCACAATACAGAGTGGGGTTGGGAGGGTAGATGTTATCATACACATGGCGGCCCAGCTACATTTATGGCA<br>GTTATTGCTAAAGGAAAAATACCAATAGTTGTTCCGAGATTAAAGAAGTATTGGTGAGCATGTGAATGATCATCAGATGGATTGATACAACCCATGGCGACTGAAATGTCACTAGGAGAAGATATGTCATTCGTTTGCCAATACTTGATGGTCTCAGAGTATTGCAGTAATTGATGGACTGTATT<br>ATACCATGTGCCATCCACAGTTCGTAAAAGATACTGGCTGAGGAGCTATCTATTCAGGATAAGGTCTACTTTGTGGGAGTTCAGTCGAATATATCAGAATGGTTGAGTACTTTTGACTCCGTCTGCTGATGATTGCGCATGGGTACATCTGTTCGCCATAACTTCATCTTAGCACCACAAAACTTC<br>TCATTCTTCGAGCCTAATTATTAATCACCGAGATAGAAGCGTAAGGGGGTACTAGAGTAGATCGACAATGGACATTTTCCAAATGAAGATGTCCACCTTATATCCAACAGAACGGTAGATATTTTGATTGTGCCGAAAGGTTTAGGAATAGCTGCATTAGAGCAACTGGTATTTTTGATATTGGA<br>GAACAAAATGCTAACTTCGTTGGAGGTGTATGGCATGGCATTCCTTTGGGATTCTTGTTACCGTTAATTAGTTTGCTACAGGTTTTAGTATTGAGAAAGCGGATGATTGTAGCGATCCAACTATACCTCTTATATTGCTTTATGAATACTATTACTGGAACGCCTGTTGTTATTCACGCCTTACGTA<br>ACCGTTGGCCCTTGTGGTGGATGGAGTAAGGCTATTCGAGAAGTGCAACTTGAAGATGAAGCCTGTGGTCATGCGAGATACGACAGAAAGACCTGAAGGAGTAGCTGCTGGAACGTTGAAATTGGTTGGGACTGATGAGGAGACTAAATCACTCACAAAGAAAATTTAACGCTTGATGTGATG<br>ACCCCATAGTGTTCGAGGTCATTCCGAGTTATCCAG
A3	GATGACCTTCGTGGCGTGCTAGTATGCATAGTGTTCGAGATGACCTGCTTCATGCCATGTGGTCCAGCTACTTTTATGGCTACTCCAATAGTTGTTCCGAGGCAAAAGAATGGGGAGCATGTGAATGATCATCTTGGAGTTGCTAATTATGGGAAGTCTGCGGGTATTTTACGATATGCTGTGCAG<br>AGTGTGCACGTGTAAGAGATGATGAGGCATGCATATTAGTCAATGCAACTACAGGTATTTTTGATATCGGAGTAGAAAATAGTAGGTTGATTGGCGGTGCTCAGCGCTTCACAAACGTAGTAAACTAGTGAGTACGGGGTCTAGGCATCTGATTATGTCATTCGATTGCTTGGAACCTACGGTAA<br>CCCACGCCCATAATGAGCTTTTCAAACAGGAATTTTAGGATTTGCATATTGTTGGATATAGGTGGTCGTATATACTTGATGAATAAATCGGATTATTCGGACGACCAGTTGAACAGACCTATCTGTATGTTGATAATTTGTATGTCTCGCTCTAGGAATAATGCTCCTAGTTTTTGGGAGGAAGTG<br>CTTTTACTTCTGTGCTAACAGTGGAGATATAGTTATGAATCATGAGAAAGCGGTTGCATTGATACGAAGAGAAGCATCGAAGATTATTCGCTTGCCTCTTCATGGATTTGTAAGCGGACGTGCGATTGTTCTGGAAAGCTTATTTCGGCTATTAAGGGTCTCCAGAGGTATTGTAGGTGGACTTTC<br>CGATGCAAGAAATGTCGGTATAGATAAAGCGGAGATTACCTCATTTTTGTTGACAGTGTCATGAACGGTTGGTTATGTCCAAAGCGGCGATTTGGACGGCAGAGAAGGTACTGGAAAAAGAACTTCTGGCCTGGCAGGAGACTGCATCAGCTATCCTGGAACTCTAGTGGCTGGTGCATTGTAT<br>TTTCACCAGCTGTATTAGGTACATGCTGATCAAGTGACAACTCCAGGGCCCTCGATGCTGGGAGCCGGTGGCGACAGCGAGACAGAATCCCGTGCAGATAGGCTTTTAGCTTGAGCGCGAAGTGAGCCAAGGCGGCCACATGGTTACCCATGGCGATGATGCACACTTAGGTGATTTACCTGCA<br>TTCATGATGGCACAGCAACAAATCCTGTTTTCTCTATTATGATCCACACGGGTGGCACGATTATGCTAGTCATCACCGAATACGGTGTGGATACGTTAGCGTATGCGGCCTGCA
B1	TTAACTTTAAGCTTGATGTGATGACCTTTTGCCATAGTGTTCGAGGTCATTGGTCAGCAGAAAGTATGCATTGGTTATTGGATGTGGTTTATCGTGAAGACCATCATCAGACCCTGGATAAACGAGCTGCACCTTAATCTTATCCGGTGCATTGCTAGAGATGGTTCCTTCAGTTGATATTGATAAA<br>GATTATTTATATATAGAAAAACTGGCAGTTTGCACTAGAGTATGGGAAGGTGTTGTTGAGCAACTGGTCTTGTATCGAAGACATGGACATAATGTAACAACTAGTCATCATCCAGATATAGTCATTCCCAATCAGGGGTTGGTGGGTCAGTAATCGATAATGAGGGAAAAATTCATCATGGGATT<br>TACCTTCTTTTAAGAGCGTTGTACTTGCAGAGGTTGAAGGTATCCTATATACTGCTTTAGGTATGTAGCGGTTGTCAATGTGTTTCTTTCAGATATTGGCAGATTCGGGAATAGGACCTGCAATAAAATATGAGATTGGTAAGGAATATTCTGGTCAACGGATTCCAATGCACTCGGTAACTTATG<br>AATGGAATTCGTTTAAGTGAAAAGACATGTTGACATTCAGTAGTATGGAGATACCGCATTTCTTATCGCACTATATTTTGAATCAGTCTGACCGTTTGATGATTAACATGTTTGATGGTAAGGAGAAATTAGCTATATATAGTCTAGCTGGTTGCTGATCAAAAGGTCTATGTTTCACTTTGATGAT<br>TTGGAAAGATTTTTATCTAATTGCTAAAAATCTTACTTTGAACCTAGGATATGCTGAACGATATGGATCGGATTTAGTAGTAACCCCATTGAGAGTAGTAAGAGATCATATTCAAGGTGGAAATGCATCCTCAATCAAGAATGCCATTATAGGGGAGAGATCTTGAATCAGGTAGATTGATTTCT<br>GCTATGTCTCCGGCTTTTGTCTTCTCTGTATTAGGAAATATCATTAGGACTCAGTATTTGATTCCTAGAGGATGTTAGAACTATGTGGGATGCTAGCAGGAACTTTTCTAGGGGCTATACGCCATAATGGATTCATCCCGTGGGACGTTTCGGATTTGGACAGGGAGACAGATCGAGATGATTATC<br>GTGGCTTCTGGATACTATGTACGAACAGATGGACTTGACCGACTATACTCCATTTGAGACGTTTATTCGGGCGAGTTAGTGAGTTGTTGTACGCCTTATGTACATGGCATTTGCAGGATTTCTAACTCTGATTGAATACTTGTATGCTTATGGTCTTTTCGATTAACAACTCTGGACGTTAGATTTTG<br>CTGTCAGCGACGTATTTACTATGAGTAAATGGTTTTCAGATTACTTGATAGCTCTGAGTTCATTATGTGGGGGCAGGAACAAATCTTGACCACTCTCATAAGGAACGTAATGAGACATGGACTTACTATAAGACTTGACAGTAGAAGGAAAAGTCAAATCTGTCTCTTAG
B2	ATCTTCCTACAGGAAGGACGAAGCCAATATACAACCTTGTACGTCTTAGTAATGGATATACAGGTTCGGGAAATTGCAACAAGAAGTGGCAGATGATTTATCATTGCTTATGGCGCAGAGATGACGTATCTGGGGTGGATTGTATAGTCTATTAAATAACCCATTGGAATTGAAACGGCTTGGAG<br>AATGTGCCCATAAGCGAATTACGGAACAGTTTATGTGGTCTTCAATTGCGAGGAGATTTGATTGAGGCAGACATTGCTATTTGCGATCCTGTTCATTCTTTCTCCGGCATCAAACGTTAAGGTTTACGAAACAACGGAGGCTCTATGTGAGATGTTTTATCACCGCTTATTTATGATACCAACTAG<br>AATCAGCAGGACACCCGCTGGACTTACTGCAATACTGCGAGTAAATTATCAAAAGATATTCCATTTTTGAGGTGCGCTATTCCCTGTCACAGTCAGAGACAGTTTCAGCAAATCTTAGATGGAAGACAAGTTTGTGGATAGGACACGGTCCAACGTTTATAAGAGGTGTAGGCTATGCTCAGGGA<br>GGATTATTTCTATTTCTAATCAAGGCATGGAGGTGGAGCATACAAGACAATTTAAACAATATTAGTAAAGCGCAGGTCAAGCAAATATTTTATATCGAGTTGTGCAGTCTTTACAAAGGTGCCTGGTTTGGCAAGTCGAAGTTTTTATATCTCTATTGGTTTGTAATCATTCTTTCTTCAATTCCTT<br>TCTGAGCTTCGTCCAGTTCCTGGGATATTAGTAGCGATGCTGTGAATTATAGTAAAGAGGAATTGGTAGAGAGTTTGTGGATCCACTTGATGGAATGCCTGCTGCCCCGACGGATGATGGTTCAACTTTCTCAGTTTAATAGTTTAGTAAAGCAGGTGACTGGTAGGTAACACAGCAGTTGCTTGC<br>GGTGCACTTGTAGGAGCAGCATTAGGAGCCACTGGTCTCTTAGGAACTGTAACTTTGACAAATATGGAGCAAATGATATTTTACCTGGTTTAAC
B3	ACTGTGAAGAAATACTCTTCGTATGATGTTGATCCAGACTATGTGACTCGACAGAATTTTCTGAATTAGGTGATTATCTTTATGAACAGGGATGAAAGCTCGGCTTGCATTTCTCTTTCTGTTGAATTTGATTGTCGCCAACAAACTATTGACAACTTCTGTTATTTTGCCCGAGAACCATTGTATC<br>GTATTGGAGCGGACTAAAGCAAATATAGAACGTGATAAGAACCGCCAAGCCTCATGAGGCGCTGACGGCGGTGAGTGCCGCTTTTACAACCACTGCTTTTGCAGCTGTTAGCCAGCCTGTTGTCTTGGCAGGCGATACAGTGGTCAGTGATCGCCGCACTCGCTACTATTTCTTACCTCAAATCC<br>GCAACTCATCAAGGATTTCTGTTACCAACTATTACTGGTTTCCAAGACATTGTGACCGGGCAATACGCAGCTACTAGTAGCTCAGCAAATGCATCACAAACAGATAATGGCGTAAATAGGTGGTTCTGAAGATCCAACAGTATATAGTGCTGAGAGGATGGGACACGGTCCCTCCTACGGGAGG<br>CAGCAGTTGTGCCAGCAGCCGCGGTAATACGGCAAACAGTAGATACCCTGGTAGTCCCATCAAGCATAAGCCATATAAGTTTTGCAATCATTGGTGGCTTAGTAAGTGCAAGCTGTAAGCGGAAAAGCACTTGCCTTATCAGGAACTTGTGTTAGTATGTAAGGTACCTCATAACTGCCGCCAGC<br>TCGTACTTCGACAATACGTCGACACTTATGGCGATAAATGCCGGCCGTATCGGATCTTTACCTTGCGATGCGTAACTGCCGTTTCGAACGCGTGGAATGGCGAAGACCCAGGGCGCACGCTGTCCGTTCTATTTGAAGCCAACGGCCTGCAATCATCCGATGTACGTGGCCAAGGCCGAACGCTT<br>CATAGTTACGCTCACACCTACCAGAACTCAACAGTCGGCCTTGCGTGAGTGGGATGCTCAAGAGCCTGCTAAACCAGCAGATACGAAAGAAGCATCAAAGGCTGCTGTTGCTAGAAAGATCGCTTGGAACCTTTTAAGCACGCCGTAAACGACAATCAATACCATATTCCTTATTTAGAAGCCA<br>GTATTGAGGCTC
C1	GATATGATAGTTGATGGAGACAGGTGACATATGACTACACTGTATCAAGTTGTTCATATATTCGTTATTTAGTGAATTGCTATACTCCGATGTTGGCAGCAGCAGTAATGTAGATTCTTATTCTGCAGGGAGAGCTCACATTAGTTTAATGCTCAGCGATAGCTTTCTCCAAGTGGAAGACCCCAG<br>CAATTCAAGTGTTCGCGGAGCGGTAAACGATTTGTTGGCTAAGTGGCATCAAGAAATGGCCGCTATCAAATTTGGCGTATTGCTTATCACTTCCGTGGCAAGATTCTGGTGCTAAGATGGGGATCAACTATGCTCATAATCAGCATGCCAGATGTACCTTTGTCGTATTGGTTTCGGACTCTTTCG<br>TGGATGGTTGTACGTGGAATCGGATTTGGTCCGCATTGGACAAGGAGTTTCTATCTTAGCAAAGCGTAAATTATTTGTTGGCAACTAGTAAAGATTTCATGTCCTATAAGGGAGAAGTTTGTACATGGTCTGGATTTATGATTGCTTCTCATAAACAGGGACGCCTAGTTAGTATCATCACCAGAT<br>ATCTTTCGACGACGTATCAGCTTCACCACCCAAAACGGTTGACGCATTATACTTATGAAGAGGTTAAGGCTCTCAATGACAATTAGCAATAGATAATGGAGTTGATATGCCATTTGAAGGACCAGTTGTTGAAATGGCATTGCTTCGTAAAGTTGGTATTCCTGATAGTGGCTTTTTTATTGTAAT<br>TGGTATCGTCTCTTTTAGTGTTGAAGAAATTGCTCCAGAAACATATAGAGCTATTTATCTTTCGTGGTTTTTGTTAGTGGTTGAGGCTAGTCTTTTGGTTTATATTAATTCCATTTCTGAAAGTAATTAGTAAGATTAACGGAAGATTGGTTGCTGACAATTTTGGGACTGTGGATGTTGGAGCTATT<br>TTAAAAGTAGATGGAAGCAAGTCTTACGTAGAACCTCTCTGGATGAATTGCCACAGATTTGGAATATTTTGGTCGGTCACATGTCGCTTGTAGGACCAATAATAGGTAGAGGAAGTGATTCATTAGCGATTTAGCGACTGATATCCTATGTGGAAGGCGTTTGAAGGTGACTATCACGCGAGATC<br>GATTTTGAGAGACTATTCGAGCAATTGGACGACTTCGAGTTGTGCCGTTTTTACAAACGCAGAGAAAAGACTAGGATTCAGAAAAATTTTAGAGGTGGGTACGCCTTGGAAGATTCGCATGAGAAATGACGTCCTCATCAGAGAGGGATAGCAAGTTATTGTCGGCG
C2	TGATTCAACTGTTCTAATTACCGCTCCAAGAGCATTTACAAACATAGGTCGGTCATTTTATTAGCAGAAGTTTTAAGTCACGGACATCCTTGCGGTTTTACGTGGGGGGACCCTTAGTGACATCTGTAATGCTGAGAAGGCTCTCGGAGGATTGTGTACTTCCTGTTCTAGGCTTGGTGGAGCAGT<br>GGTTGGGTTTGAACAAGAGAGACGCTATTTTCCTCTGGGAAGTGGTTTTGCTACATCTGGAACTGGTTCAGCAACTATACGCTGGCATGGCAGAAAAGTCCTAAGGTATTTCAGACGGTCTTAGAACCTGTCCAATGAAGAGCAAGACTTGACAGTCCTGTTAGTGCACCTGTATTTAACGATAA<br>ATGGCGTGAACAACGAATAGGAGATGAAGGAGTTTTGAATGCTGTTCTGGAAGGACATGTCTTGATTGTCCGGTGGAGTAGGTCAAGATGGGAACTATTCAAGTACATTGGCGCTCCGTTACTCAGTATATTGGGGAGAACAGACACCCTAGCTATGGGATGAACCTGTGTTTCAAGCTTCCCCT<br>TTAGACTTTCAAAATACCCAGGACACCCGTTCAACGATACGTGATACTTTGATTTCATTTTTTGTTATGGGTGGGGAGATGAGATATTGATCTAGCTACTTGACTAAAATATTTGAACTGCTAGGGAGCGAGGTCACTAAAAGTCATACAGCGAAATCGACTCGTCTACCAAGATAGGGAGAGCA<br>GCATTAGGGGCAGGTTTTGCTTCATATGGGAGCAACGCTGCTTATGTGTATTACTCTCCCCTGTAGCTAATGATAACTTTTGGCCGATTTTTGAGCAGTTGCTCCTCTATTTGTACCAACGGGGCGTGCGTTATGGACTGGCTGATGGTTCTCTAGGTCAACGTATTGGAACTCTTAGAAATTGGGA<br>AAATAAAGGCTATTATTGGGTCAGGATTTATGCACAGTTTGGTGGTTCTATATCCTCTCCTATCAACTATTACTCATTATACTACCTATCATTACAACTCCCTATGTGACACGGGTCTTTTCTTTGAATGATCTAGGGACTTATGGTTATTCTGATGTTCATGGCTATTATGAAGCGTGGAATTTTCT<br>ATACTGCAATAGGGCAATTCTTAGCCGGATTCTCTCACCTGTAGAATATGGTATCGTAGCAGTAGGATTCAATGTACAAGTGGTATCATTATAGTGGGGGTGTTATACGGCGAAACTATTTATGTTAGTTTTCCGC
C3	GAGCTCATGTCTAAGTGAAGTGGTCGATGGTGGGTCCTGTTCCGCTATTCCATCAAGATTTAGCTCGTCGTATTGGACTTTGGCAATGCAGATATCGGTACCCAGAAAATTACTCCTCAGCAAGAGGCACAGTTTGCTCCATTACAACCCAGGCCGAATATGACGAATTAACTACCGTCACCCGCC<br>ACAGATTCATGAGCAGATCACCCGTCTGTTCCACGCGTTAGTGCGTTAAGTACTCCCCTCCATTACCCATTTGGTATGGGAGTAGGGAGTGTGGGAGAGGTTCACAAACAGCACTTTCTTCAGAGGAGGAATTGGAATGATAATTGCAACACTCAGTTTCGTTTTATTACCAAGCGCATTCTTCTT<br>CGTCCATAACCTTCTCTAGCCTTGTTGTTACTGGAAACTGATCATAATGCGAAATTTGCTCGCCATACTGCACAATTGGTTTAACCGCAGGCAAAGCATTTAATCTTGGCAATTTTGGGGTTACACAGACATCGACACACACGAGTACATCTACCATCACATCTACGATTACATACTCACATCAAC<br>GAGGCGGTGTAGCAATGGTTATTTCGTTCTTTGCTAATCAGGCAGACTGGCCTTGTCTATTCAGCCCTTCTGGTCTCATTGTAGCGGGCATTCTTGAGTTCCCAATTTACAATATAATCGCGAAGGAGAACATAACTATGGAGGACAAAAAAATGAACTAACTCAAATAGTAACGTAAGTCAGGC<br>GTATTCTTCACAAGGGAAATACCGCAAATAAACAAGAAAATTACAGTATGTATCTCTTAAGGTTGAATAGAAAGTTTTGAGCAAATAGATGTATCCCAGTTAACACTACCTTTGAAGCTAGACTATTCGGTCGTGGGACATTAAACCAAAAGAGCTACCGAAGTTGAACTGCGAATATTTAAAG<br>TTGAAAGAGGCTACATCAAATAGTTGGCGAGCTCGGTAGGGGTGAGTACTATTGGAGTGCTTTGGAAGTTACGGTGTGATTCAGTAGTAATTAGTATGGGACAGTTGGGATGATTGGTCGTATTAGTAGATGGCGAATTGCGTGGATCGAACTGAAGAACTAATTGAAGAGGTACATTACGCCA<br>GACCGCCATGGCATACAGTGGTTACTTACGAAAGTATAGGCTTTTGGT

Table S3: Assay-set pools for specific target amplification/Pre-Amplification (Pre-Amp)

**Pool A (n=32)**	**Pool B (n=32)**	**Pool C (n=34)**
1	3	2
4	6A/C	6C/D
5	7B/C, 40	9L/N
6A/B/C/D(F/G/H)	9A/V	10A
7A/F	10B	11B/C
8	11A/B/C/D/F/(E)	15A/F
9A/L/N/V	12B	16F, 18F, 28A/F
10C/F	13	17F
11A/D	15A/B/C/F	19A
11F	16A	21
12A/B/F, 44, 46	34, 37, 17A	23A
14	18B/C/F	23B
16F	19F	24B/F
15B/C	22A/F	25A/F, 38
18A/B/C	23F	27
19B/F	25A/F	29
20	33C	31
22F	35B	32A/F
23A/B/F	46	33A/F, 37
24A	47A/F	33B
28A/F	Pia*B*	34
33D	Bex*A*	35A/C, 42
17A	IS481	36
35F, 47F	Ptx*S1*	39
41F	pIS1001	41A
Eco	*Mca*	43
Lyt*A*	*Nla*	45
Sag	*Sau*	48
Hin-b	*Spy*	IgA1
hIS1001	Xisco	Ply
Nme	16S	Bex*B*
-	*Sor*	*Pji*
-	-	*Aba*
-	-	*Kpn*
